# Neural correlates of repetitive negative thinking: Dimensional evidence across the psychopathological continuum

**DOI:** 10.3389/fpsyt.2022.915316

**Published:** 2022-07-22

**Authors:** Jasper van Oort, Indira Tendolkar, Rose Collard, Dirk E. M. Geurts, Janna N. Vrijsen, Fleur A. Duyser, Nils Kohn, Guillén Fernández, Aart H. Schene, Philip F. P. van Eijndhoven

**Affiliations:** ^1^Department of Psychiatry, Radboud University Medical Center, Nijmegen, Netherlands; ^2^Department of Cognitive Neuroscience, Radboud University Medical Center, Nijmegen, Netherlands; ^3^Donders Institute for Brain, Cognition and Behavior, Radboud University and Radboud University Medical Center, Nijmegen, Netherlands; ^4^Pro Persona Mental Health Care, Depression Expertise Center, Nijmegen, Netherlands

**Keywords:** fMRI, network, connectivity, RNT, rumination, worry

## Abstract

Repetitive negative thinking (RNT) captures an important transdiagnostic factor that predisposes to a maladaptive stress response and contributes to diverse psychiatric disorders. Although RNT can best be seen as a continuous symptom dimension that cuts across boundaries from health to various psychiatric disorders, the neural mechanisms underlying RNT have almost exclusively been studied in health and stress-related disorders, such as depression and anxiety disorders. We set out to study RNT from a large-scale brain network perspective in a diverse population consisting of healthy subjects and patients with a broader range of psychiatric disorders. We studied 46 healthy subjects along with 153 patients with a stress-related and/or neurodevelopmental disorder. We focused on three networks, that are associated with RNT and diverse psychiatric disorders: the salience network, default mode network (DMN) and frontoparietal network (FPN). We investigated the relationship of RNT with both network connectivity strength at rest and with the stress-induced changes in connectivity. Across our whole sample, the level of RNT was positively associated with the connectivity strength of the left FPN at rest, but negatively associated with stress-induced changes in DMN connectivity. These findings may reflect an upregulation of the FPN in an attempt to divert attention away from RNT, while the DMN result may reflect a less flexible adaptation to stress, related to RNT. Additionally, we discuss how our findings fit into the non-invasive neurostimulation literature. Taken together, our results provide initial insight in the neural mechanisms of RNT across the spectrum from health to diverse psychiatric disorders.

## Introduction

Stress-vulnerability models provide a transdiagnostic framework to explain how psychopathology arises from specific interactions between stressors and individual vulnerabilities (1). An individual's tendency to Repetitive Negative Thinking (RNT) captures an important transdiagnostic cognitive vulnerability, that predisposes to a maladaptive stress response, and thereby constitutes a risk factor for a broad range of psychiatric disorders (2, 3). RNT entails a way of thinking about problems or negative experiences that is repetitive, intrusive, and difficult to disengage from (4). Initially, research on RNT started in disorder-specific research, using the concept ‘rumination' in relation to depression and ‘worry' when studying anxiety disorders. Recently, however, it has been recognized that worry and rumination are in fact both characterized by the same core elements that are mentioned above (5).

RNT can best be seen as a continuous symptom dimension that not only cuts across diagnostic boundaries (6), but also from health to disorder (7). In health, it is an important risk factor for developing stress-related disorders (i.e., depression and anxiety disorders) (7). In psychopathology, it reflects a crucial transdiagnostic factor in the onset, maintenance, and recurrence of especially (comorbid) stress-related disorders (6, 8, 9). There are however clear indications that RNT is present in most psychiatric disorders (4) with also a high prevalence in neurodevelopmental disorders [i.e., autism spectrum disorder (ASD) and attention deficit hyperactivity disorder (ADHD)] (3, 10, 11).

At the psychological level, RNT may be explained by the impaired disengagement theory, which states that RNT results from impaired cognitive control to divert attention away from one's negative thoughts (8). In health, negative information about oneself is often in discord with positive self-views and leads to cognitive conflict and disengagement from these thoughts. Pre-existent negative cognitive schemas may predispose someone to RNT and the development of a stress-related disorder, by preventing the employment of these self-regulatory strategies (3). Patients with neurodevelopmental disorders may have specific predispositions, which make them vulnerable to RNT. Patients with ADHD are prone to worrisome, intrusive thoughts due to cognitive control deficits (11) and common traits in ASD, like heightened self-focus, and cognitive inflexibility in attention switching, may predispose these patients to RNT (3). This vulnerability for RNT in neurodevelopmental disorders may be a key factor explaining the high comorbidity with stress-related disorders (3).

Despite the important role of RNT as a transdiagnostic factor in psychopathology, the mechanistic underpinnings at the brain level are still unclear. Three large-scale functional brain networks, revealed by functional magnetic resonance imaging (fMRI) (12–14), have been implied in the underlying pathophysiology of RNT, and are largely consistent with the impaired disengagement theory (2, 3, 8). The frontoparietal network (FPN) due to its role in top-down cognitive control (15, 16), the salience network (SN) for its role in flexible attention shifting (14) and the default mode network (DMN) as the neural correlate of self-referential processing (17).

Interestingly, the resting-state literature has shown a key role of these three networks in depression and generalized anxiety disorder (GAD), in which rumination and worry are core symptoms (5, 14). The meta-analysis of Kaiser and colleagues (18) and various other studies have shown decreased connectivity within the FPN in depression (19, 20). While the meta-analysis of Kaiser and colleagues showed increased connectivity between DMN regions (18), the meta-analysis of Tozzi and colleagues revealed reduced connectivity within the midline core subsystem of the DMN (21). Reviews have suggested that depression is characterized by increased connectivity within the anterior DMN and by a dissociation between the anterior and posterior DMN (19, 20). Furthermore, there is evidence for decreased within DMN connectivity in GAD (22). Although there is more evidence for decreased connectivity within the SN in depression, there is also some conflicting evidence suggesting increased connectivity between SN regions in depression and GAD (19, 23). While these studies underscore the key role of these three networks in depression and GAD, there are also sometimes contradictory findings with respect to the exact role of these networks. This may be related to limitations in the performed studies, like small sample sizes (19), but also to the heterogeneous nature of these traditional psychiatric classifications (24). This emphasizes the importance of specifically studying the relationship between these networks and core symptom dimensions like RNT.

Importantly, the resting-state studies that specifically investigated RNT across the population, from health to psychiatric disorders, have also highlighted the importance of our networks of interest in this symptom dimension (3, 25–27). In their landmark study, Hamilton and colleagues (25) showed that in depression RNT, in the form of rumination, was associated with a relative dominance of DMN over FPN activity during rest. They also showed the importance of the SN in dynamically switching between the DMN and FPN (25, 28). Since then, resting-state studies have shown a negative relationship between RNT and connectivity strength within the DMN in generalized anxiety disorder, depression and health (29, 30).

Although these networks also have a central role in the acute stress response (31), only few studies have investigated the relationship between RNT and stress-induced network changes. Rosenbaum and colleagues (7) have shown that in healthy subjects there was a higher increase in stress-induced FPN activity in low ruminators compared to high ruminators. Lydon-Staley and colleagues (2) showed that higher connectivity between the DMN and FPN during sad mood induction, in health and remitted depression, predicted increased RNT. In short, there is ample evidence for the importance of these networks in RNT, both at rest and in response to stress/mood induction, but the literature is largely restricted to specific populations (mainly health and stress-related disorders) (26, 27).

In the present study, we, therefore, set out to investigate RNT as a transdiagnostic factor in a population with both healthy subjects and patients with stress-related and/or neurodevelopmental disorders from a functional network perspective. For our primary question, we studied how the connectivity strength of our networks of interest, both at rest and with respect to stress-induced changes in connectivity strength, underlies (content-independent) RNT across the whole sample. Based on the literature described above, we hypothesized that RNT would be associated with decreased connectivity within all three our networks of interest. Secondarily, we investigated whether there were specific differences between subgroups (healthy subjects, stress-related disorders, neurodevelopmental disorders and a comorbidity group) with regard to the relationship between RNT and the connectivity strength.

## Material and methods

### Subjects

This study is part of the MIND-Set study (Measuring Integrated Novel Dimensions in Neurodevelopmental and Stress-related Mental Disorders) (32). The MIND-Set cohort includes adult patients with a neurodevelopmental (ASD and/or ADHD) and/or stress-related disorder (mood and/or anxiety disorder). A psychiatrically healthy control group is also included. Patients were diagnosed by a trained clinician according to the Diagnostic and Statistical Manual of Mental Disorders (DSM) (33) using semi-structured interviews (i.e., the Structured Clinical Interview for DSM-IV Axis I Disorders (SCID-I) (34), the Diagnostic Interview for ADHD in Adults (35) and the Dutch Interview for ASD in Adults (36)). The MIND-Set study has been approved by the Ethical Review Board of the Radboudumc (Nijmegen, the Netherlands) and all participants signed informed consent before participation. In the present study, we included the subjects from our earlier study (37), for which the RNT score was available. We refer to this earlier study for an elaborate description of the study procedure.

While the primary analyses were performed in the combined subject group, for additional secondary analyses, related to potential differences across the diagnostic spectrum, this group can be considered as consisting of four subgroups:

1. Healthy control group: subjects without a present or past psychiatric disorder.
2. Stress-related group: patients with a current depressive episode, dysthymia, and/or anxiety disorder, but no ADHD or ASD.
3. Neurodevelopmental group: patients with ASD and/or ADHD, but no stress-related disorder.
4. Comorbidity group: patients with both a stress-related and neurodevelopmental disorder.

### Procedure

**Figure 1:**
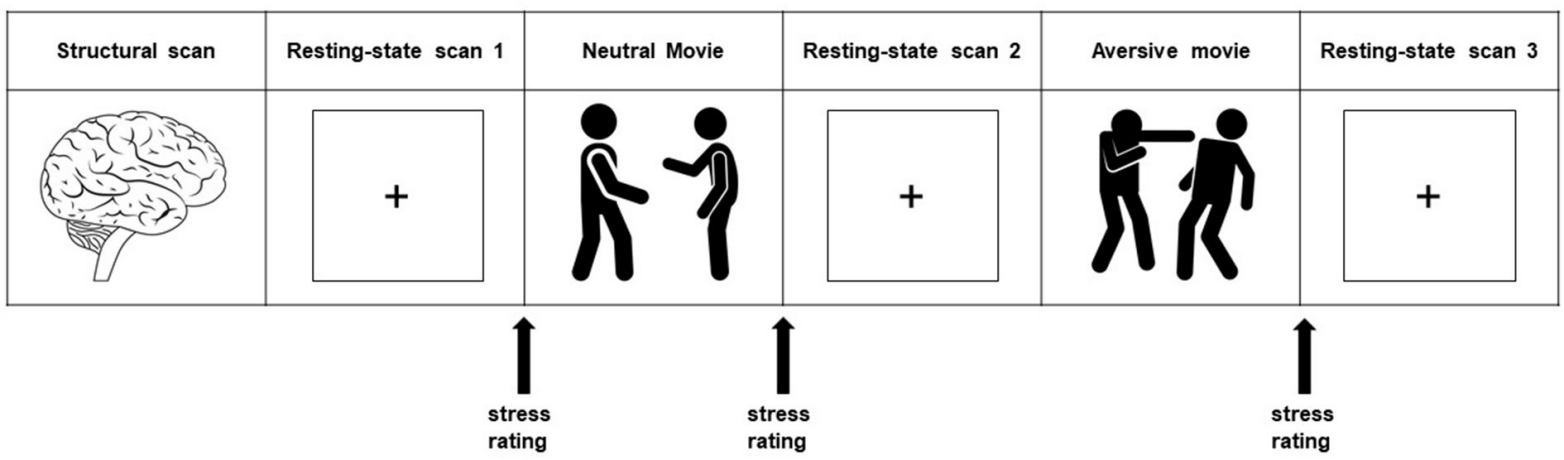
Experimental design. The whole protocol consists of a series of scans of which we used the structural scan and the three resting-state scans (8:30 minutes each) for our present study. Resting-state scan 1 is used to study the brain at rest, and is referred to as the baseline resting-state scan. Stress induction took place with a highly aversive movie clip (2:20 minutes), with a self-referential eyewitness instruction. The neutral movie clip (2:20 minutes) served as a control condition. Stress-induced changes in connectivity strength were investigated using the resting-state scans directly after these movie clips (stress minus neutral: resting-state 3 minus resting-state 2). Subjective stress levels were assessed with an 11-point rating scale. Heart rate (in beats per minute) was measured continuously during scanning.

To ensure a relaxed baseline state, the subjects had a 45-min pre-scanning acclimatization period. For the primary analysis, we investigated the brain's functional connectivity during a baseline resting-state scan. This is the first of three resting-state scans in our scan protocol (i.e., resting-state scan 1). Additionally, we studied the brain's response to a mild psychological stressor using an experimentally well-controlled paradigm in the form of an aversive movie clip (38). This stressor consisted of a movie clip (140 s) from the movie ‘Irréversible' (2002), by Gaspar Noé, and showed extreme male-to-male aggression. Participants were asked to constantly and attentively watch the movie clip after a short introductory text put them in the scene from an eye-witness perspective, in order to maximally involve them in the experience (38, 39). A neutral movie clip served as a control condition. We studied the brain's stress response by using the resting-state scans directly after these movie clips (Figure 1). The neutral movie clip and its subsequent resting-state scan (i.e., resting-state scan 2) always preceded the aversive movie clip and its ensuing resting-state scan (i.e., resting-state scan 3).

### Questionnaires

Demographic information (age, sex, and level of education) was acquired using a questionnaire from the Dutch Helius study (40). The level of education was measured as an ordinal variable with four levels (41).

The Perseverative Thinking Questionnaire (PTQ) was used to measure trait RNT. The PTQ is a 15-item self-report questionnaire that assesses trait RNT from a transdiagnostic perspective (i.e., independent from disorder specific content), in both clinical and general populations (4). Items are rated on a 5-point scale, from 0: “never” to 4: “almost always.” We used the PTQ sum score for our primary analyses. Secondarily, we also used the following three subscales of this questionnaire: core characteristics, unproductiveness, and capturing mental capacity. These subscales have been identified in previous factor analyses (4) and capture different aspects of RNT. The core characteristics subscale measures the repetitiveness, intrusiveness, and difficulties with disengaging from these thoughts (e.g., “The same thoughts keep going through my mind again and again”). The unproductiveness subscale assesses to what extent someone perceives these thoughts as not being helpful for solving one's problems (e.g., “I keep asking myself questions without finding an answer”). Finally, the capturing mental capacity subscale measures to what extent the repetitive thoughts occupy someone's attention and if they prevent someone from focusing on other things than the problems (e.g., “My thoughts prevent me from focusing on other things”). High internal consistencies were found for as well the PTQ sum score (α = 0.94–0.95) as all three subscales (Core Characteristics of RNT: α = 0.92–0.94; Unproductiveness of RNT: α = 0.77–0.87; RNT Capturing Mental Capacity: α = 0.82–0.90) (4). Finally, results have also shown a satisfactory test-retest reliability for the PTQ sum score (*r*_tt_ = 0.69; *p* < 0.001) and all three subscales (Core Characteristics of RNT (*r*_tt_ = 0.66; *p* < 0.001), Unproductiveness of RNT (*r*_tt_ = 0.68; *p* < 0.001), RNT Capturing Mental Capacity (*r*_tt_ = 0.69; *p* < 0.001)) (4).

The Inventory of Depressive Symptomatology self-report (IDS-SR) questionnaire (42), consisting of 30 items, was used to assess the severity of depressive symptoms in the past 7 days.

### fMRI acquisition and preprocessing

All images were collected using a 3T Siemens Magnetom Prisma MRI scanner (Erlangen, Germany). High-resolution structural images (1.0 mm isotropic) were acquired using a T1-weighted MP-RAGE sequence (TE/TR = 3.03/2,300 ms). T2^*^weighted EPI BOLD-fMRI images were acquired for the resting-state scans, using a multi-band 6 protocol (TR = 1,000 ms, voxel size = 2.0 mm isotropic). Preprocessing and statistical analyses were performed on the three resting-state scans (each 500 volumes) using FSL 5.0.11 (FMRIB, Oxford, UK). These scans were preprocessed using the FMRI Expert Analysis Tool (FEAT), which is part of the FMRIB Software Library (FSL) (43). To allow for T2^*^ equilibration effects, the first five images of each resting-state scan were discarded. Furthermore, the preprocessing steps included brain extraction, motion correction, bias field correction, high-pass temporal filtering with a cut-off of 100 s, spatial smoothing with a 4 mm full width at half maximum (FWHM) Gaussian kernel, registration of functional images to high-resolution T1 using boundary-based registration and non-linear registration to standard space (MNI152). The final voxel size for group analysis was 2 mm isotropic. ICA-based Advanced Removal of Motion Artifacts (ICA-AROMA) was used for further single-subject denoising (44). Subjects were excluded from analyses if motion resulted in more than 2 mm sudden relative mean displacement or translation [see also van Oort et al. (37)].

### fMRI analyses

**Figure 2:**
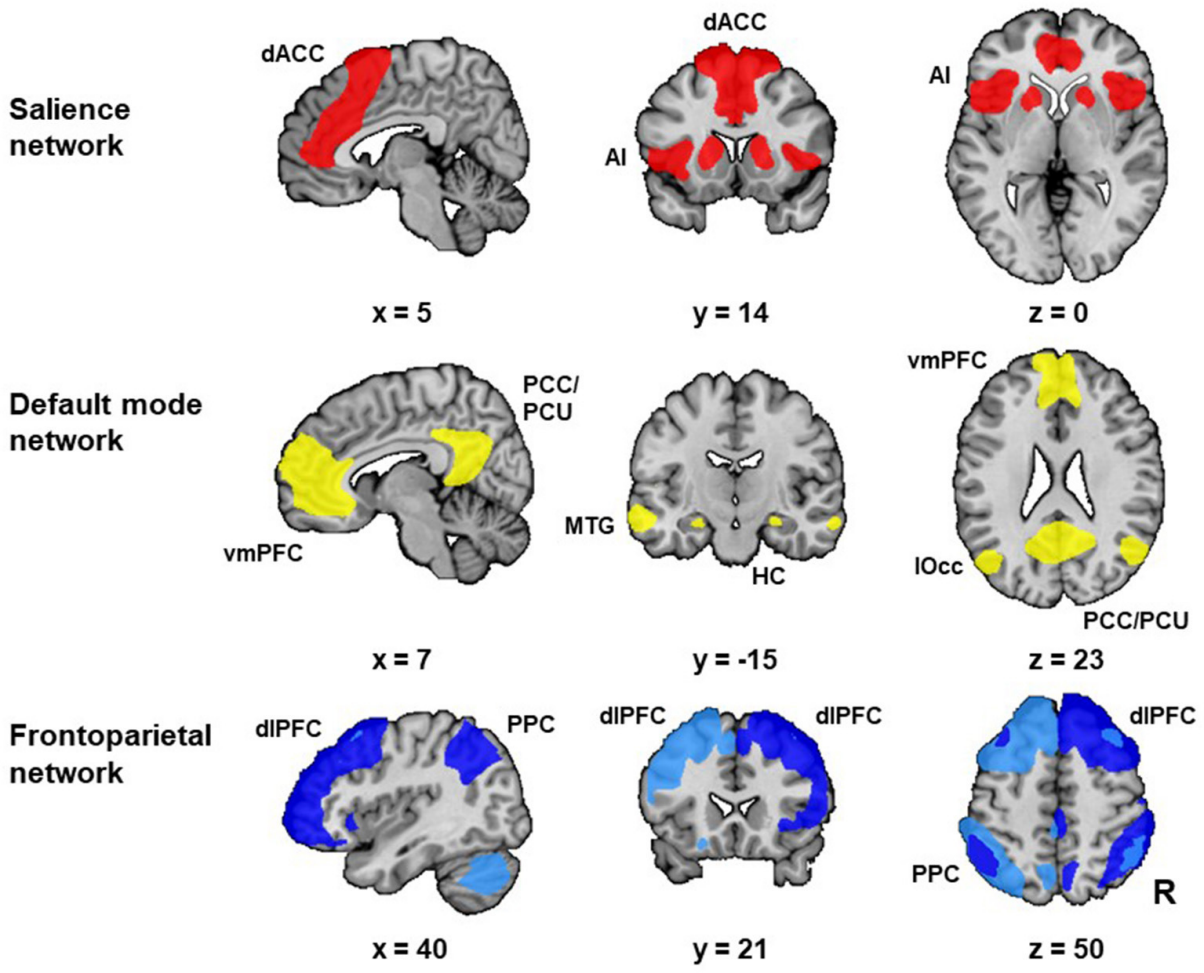
Networks of interest. The networks of interest [i.e., the salience network (SN), default mode network (DMN) and frontoparietal network (FPN)] were identified with group independent component analysis (ICA) in resting-state scan 1. We identified two FPNs: the left FPN (light blue) and right FPN (dark blue). The selected networks included all areas that are typically considered as core regions in these networks. The SN included the bilateral anterior insula (AI) and dorsal anterior cingulate cortex (dACC). The DMN included the ventromedial prefrontal cortex (vmPFC), posterior cingulate cortex (PCC) and precuneus (PCU). Additionally, the DMN included the hippocampus (HC), middle temporal gyrus (MTG) and lateral occipital cortex (lOcc). Both FPNs included the dorsolateral prefrontal cortex (dlPFC) and posterior parietal cortex (PPC). Abbreviation: R: right. [Figure adapted from van Oort et al. (37)].

We identified our networks of interest (i.e., SN, DMN, right FPN, and left FPN) in the baseline resting-state scan (i.e., resting-state scan 1). Group independent component analysis (ICA) (12) was used to decompose the data of this baseline resting-state scan of all subjects together into 20 components. Spatial cross-correlation of the unthreshholded statistical maps of our group ICA with the unthresholded statistical maps of templates from the literature was used to select the respective group-specific network components [see Smith and colleagues (13) for the DMN and FPN, and Shirer and colleagues (45) for the anterior SN template]. We selected the ICA derived components that showed the highest spatial cross-correlation with the independent network templates from the literature (13, 45). Visual inspection by two authors (JvO and PvE), confirmed the selection of the best fitting networks and the inclusion of all areas that are typically considered as core regions of these networks (i.e., for the SN: dorsal anterior cingulate cortex and anterior insula; for the DMN: the ventromedial prefrontal cortex (PFC), posterior cingulate cortex and precuneus, for both FPNs: dorsolateral PFC and posterior parietal cortex; see Figure 2 for the selected networks of interest). Next, we thresholded (*Z* ≥ 3) the statistical network masks of our networks of interest and extracted averaged network connectivity strength values for each network, from the individual spatial maps generated with dual regression (46). This approach results for each of our three resting-state scans in one value per subject and network, which represents an aggregate measure of mean within network connectivity strength, with higher values representing stronger within network connectivity [see van Oort et al. (37) for a detailed description of this procedure].

Our aim was to get a better understanding of RNT from a neuroscience perspective by investigating the relationship between the PTQ sum score and the connectivity strength of our networks of interest in the baseline resting-state scan (resting-state scan 1) in the combined subject group. We opted for a hierarchical regression model to model the effect of different variables (47). In line with earlier studies we selected the PTQ score as the dependent variable and the brain level (connectivity strength) as the predictor/explaining variable for this psychological construct (48–50). We made four separate hierarchical regression models, one for each of our four networks. In model 1 of the hierarchical regression model we included the following covariates, as these factors may be potential confounders: age (51–53), sex (52, 54–56), level of education (57, 58), IDS-SR score (25) and subject subgroup (i.e., healthy, stress-related, neurodevelopmental, and comorbidity group) (25, 59). In model 2 we included the variables from model 1 and added the network connectivity strength. In model 3, we included all variables from model 2 and added interaction terms (subject subgroup x network connectivity strength) in order to investigate potential interaction effects (for our secondary analyses). Model 2 was tested against model 1 to see if the inclusion of the network connectivity strength significantly improved model fit over and above model 1, which may provide more insight into transdiagnostic mechanisms of RNT across the combined subject group. Next, model 3 was tested against model 2 to specifically test whether the inclusion of the interaction terms significantly improved the model, compared to only the addition of network connectivity strength. This analysis may provide further insight into potential subgroup specific patterns. For the interaction analysis (model 3 vs. model 2), we decided in advance that we would use a *p*-value < 0.10 as a threshold for performing the following additional analyses: (1) test for all possible interaction effects between the four subject subgroups and network connectivity strength and (2) perform the regression model for each of the four subgroups separately. Finally, if the inclusion of the network connectivity strength significantly improved model fit (model 2 vs. model 1) or if there was a significant interaction effect (model 3 vs. model 2), then we also performed *post-hoc*, hierarchical regression models, for each of the three PTQ subscales separately. These models followed the same hierarchical structure as the regression analyses described above, in which nested models were compared against each other.

For the analyses of the relationship between the PTQ sum score and the stress-induced changes in connectivity strength, we used the same design for the hierarchical regression models as we used for our baseline resting-state scan. The only difference was that for the connectivity strength, we now used the difference score between the stress and neutral condition (resting-state 3 minus resting-state 2; Figure 1).

Finally, we would like to note that while we performed separate regression models for our different networks of interest, we did not perform a multiple comparison correction. Therefore, when interpreting the results, it is important to keep in mind the more exploratory nature of these analyses.

### Statistical analysis

We used the following two measures to assess stress levels during scanning: heart rate (beats per minute) and subjective stress (11-point rating scale: 0 = no stress, 10 = maximal stress). To assess the baseline stress level, we determined the heart rate during the baseline resting-state scan (i.e., resting-state scan 1) and subjective stress directly after this scan. To detect potential differences between the four subject subgroups, separate ANCOVA's (alpha = 0.05) were performed for both these stress measures (dependent variable), while controlling for age, sex, and level of education as covariates.

For these two stress measures, the effects of stress were assessed using a mixed model repeated measures ANCOVA (alpha = 0.05), again with age, sex, and level of education as covariates. Subjective stress was assessed directly after the aversive and neutral movie and the heart rate was measured during these two movies.

To investigate potential differences in PTQ and IDS-SR scores between the four subject subgroups an ANOVA (alpha = 0.05) was performed. Furthermore, correlations were performed between the PTQ and IDS-SR.

We performed ANOVAs to test for potential differences in the within network connectivity strength for our networks of interest (dependent variable: within network connectivity strength, fixed factor: subject subgroup). Finally, for each of the three resting state-scans, ANOVAs were performed to test for potential group differences regarding mean relative framewise displacement between successive images (dependent variable: mean relative framewise displacement, fixed factor: subject subgroup).

## Results

### Study population

In the present study we included 46 healthy subjects and 153 patients [stress-related group (*n* = 57), neurodevelopmental group (*n* = 46), comorbidity group (*n* = 50)]. While there was no significant difference between the subject subgroups with respect to sex (χ^2^ = 1.94, *p* = 0.586), there was a trend for a difference regarding age (*F*_(3, 195)_ = 2.47, *p* = 0.063), and level of education (χ^2^ = 16.88, *p* = 0.051; Table 1).

**Table 1:** Demographics and clinical characteristics.

	**Combined** **subject** **group** **(*****n*** = **199)**	**Healthy** **controls** **(*****n*** = **46)**	**Stress-related** **group** **(*****n*** = **57)**	**Neurodevelop-mental group** **(*****n*** = **46)**	**Comorbidity group** **(*****n*** = **50)**	**Test for differences between the four subgroups in the combined subject group (**χ^2^**/*****F***, ***p*****-value)**	**Comparison between the four subgroups, if AN(C)OVA is significant (statistics shown if** ***p***<**0.05) (*****p*****-value)**
**Demographics**							
Age (years), median (range)	34 (18–74)	32 (20–74)	39 (19–73)	32 (18–74)	31 (18–63)	*F*_(3, 195)_ = 2.47, *p* = 0.063	
Sex, %male (M/F)	55.8% (111/88)	50% (23/23)	52.6% (30/27)	63% (29/17)	58% (29/21)	χ^2^ = 1.94, *p* = 0.586	
**Level of education**							
No (*n*)	6	0	2	2	2	χ^2^ = 16.88, *p* = 0.051	
Low (*n*)	20	3	7	5	5		
Middle (*n*)	93	22	17	22	32		
High (*n*)	80	21	31	17	11		
**Symptoms**							
IDS-SR (mean, SD)	22.92 (± 16.25)	5.11 (± 3.74)	34.65 (± 15.15)	17.52 (± 10.74)	30.54 (± 11.97)	*F*_(3, 195)_ = 64.94, *p* <0.001**	SR > HC (*p* <0.001**), ND > HC (*p* <0.001**), CM > HC (*p* <0.001**), SR > ND (*p* <0.001**), CM > ND (*p* <0.001**)
**PTQ (mean, SD)**							
PTQ sum score	31.25 (± 12.38)	16.78 (± 7.94)	35.60 (± 11.22)	32.43 (± 9.40)	38.50 (± 7.93)	*F*_(3, 195)_ = 51.40, *p* <0.001**	SR > HC (*p* <0.001**), ND > HC (*p* <0.001**), CM > HC (*p* <0.001**), CM > ND (*p* = 0.002**)
Subscale core characteristics	19.65 (± 7.64)	11.20 (± 5.01)	22.02 (± 6.94)	20.39 (± 6.37)	24.06 (± 5.12)	*F*_(3, 195)_ = 43.16, *p* <0.001**	SR > HC (*p* <0.001**), ND > HC (*p* <0.001**), CM > HC (*p* <0.001**), CM > ND (*p* = 0.003**)
Subscale unproductiveness	5.92 (± 2.63)	3.22 (± 1.98)	7.14 (± 2.57)	5.89 (± 2.01)	7.06 (± 1.80)	*F*_(3, 195)_ = 35.57, *p* <0.001**	SR > HC (*p* <0.001**), ND > HC (*p* <0.001**), CM > HC (*p* <0.001**), SR > ND (*p* = 0.004**), CM > ND (*p* = 0.008**)
Subscale capturing mental capacity	5.67 (± 2.81)	2.37 (± 1.69)	6.44 (± 2.38)	6.15 (± 2.32)	7.38 (± 1.93)	*F*_(3, 195)_ = 51.65, *p* <0.001**	SR > HC (*p* <0.001**), ND > HC (*p* <0.001**), CM > HC (*p* <0.001**), CM > SR (*p* = 0.023*), CM > ND (*p* = 0.005**)
**Movement**							
**Mean relative framewise displacement (in mm) (mean, SD)**							
Resting-state scan 1	0.097 (± 0.052)	0.090 (± 0.045)	0.108 (± 0.071)	0.093 (± 0.042)	0.095 (± 0.041)	*F*_(3, 195)_ = 1.13, *p* = 0.338	
Resting-state scan 2	0.098 (± 0.052)	0.091 (± 0.042)	0.108 (± 0.072)	0.093 (± 0.038)	0.097 (± 0.041)	*F*_(3, 195)_ = 1.13, *p* = 0.339	
Resting-state scan 3	0.099 (± 0.051)	0.093 (± 0.047)	0.107 (± 0.068)	0.095 (± 0.041)	0.099 (± 0.041)	*F*_(3, 195)_ = 0.72, *p* = 0.541	
**Network connectivity**							
**Baseline connectivity (median, range) (arbitrary units)**							
SN	11.85 (2.76 to 31.56)	12.59 (3.34 to 31.56)	10.26 (2.76 to 26.63)	13.06 (4.04 to 30.87)	12.45 (3.67 to 21.64)	*F*_(3, 195)_ = 2.25, *p* = 0.084	
DMN	16.84 (3.90 to 31.03)	17.62 (6.53 to 29.52)	15.78 (4.05 to 31.03)	16.95 (9.38 to 29.77)	17.20 (3.90 to 29.20)	*F*_(3, 195)_ = 1.76, *p* = 0.156	
Le FPN	8.44 (0.01 to 19.02)	9.00 (0.95 to 16.41)	7.91 (1.16 to 14.86)	8.28 (1.76 to 19.02)	8.81 (0.01 to 15.87)	*F*_(3, 195)_ = 1.31, *p* = 0.273	
Ri FPN	7.93 (0.71 to 20.31)	8.72 (1.07 to 17.50)	7.43 (0.71 to 13.83)	7.49 (1.03 to 16.95)	7.87 (1.80 to 20.31)	*F*_(3, 195)_ = 1.82, *p* = 0.145	
**Connectivity change (median, range) (arbitrary units)**							
SN	0.91 (−13.11 to 24.04)	0.66 (−13.11 to 7.40)	0.62 (−6.53 to 24–04)	1.71 (−12.09 to 18.58)	0.29 (−7.56 to 12.15)	*F*_(3, 195)_ = 0.82, *p* = 0.484	
DMN	−0.15 (−11.87 to 14.44)	−1.58 (−9.49 to 5.65)	0.13 (−7.44 to 14.44)	−0.24 (−8.59 to 10.00)	0.65 (−11.87 to 8.14)	*F*_(3, 195)_ = 1.47, *p* = 0.223	
Le FPN	0.27 (−16.29 to 8.29)	0.51 (−8.03 to 5.67)	0.07 (−8.89 to 6.29)	0.42 (−16.29 to 8.29)	0.30 (−5.82 to 5.52)	*F*_(3, 195)_ = 0.57, *p* = 0.636	
Ri FPN	−0.01 (−9.51 to 12.27)	0.07 (−5.76 to 9.64)	−0.31 (−5.94 to 12.27)	−0.03 (−5.58 to 4.59)	0.24 (−9.51 to 6.52)	*F*_(3, 195)_ = 0.57, *p* = 0.636	

*CM, comorbidity group; F, female; HC, healthy controls; M, male; mm, millimeter; ND, neurodevelopmental group; SR, stress-related group*.

*χ^2^, Pearson's Chi square (2-tailed)*.

*p < 0.05.

**p < 0.01.

As expected, the scores for the IDS-SR and PTQ were higher in all patient subgroups compared to the healthy group. Furthermore, there were several differences between the different patient subgroups, with in general higher scores on the IDS-SR and PTQ (subscales) for the stress-related and comorbidity group compared to the neurodevelopmental group. The stress-related group scored higher on the PTQ “unproductiveness” subscale than the neurodevelopmental group (*p* = 0.004; see also Table 1). Finally, the PTQ sum score correlated with the IDS-SR in the combined subject group (*r* = 0.696, *p* < 0.001; Supplemental Table A).

### Behavioral and physiological results

Although there were no differences in heart rate between subject groups during resting-state scan 1 (*F*_(3, 183)_ = 1.16, *p* = 0.327), the subjective stress level was higher directly after resting-state scan 1 in all three patient subgroups compared to the healthy subjects [stress-related > healthy (*p* < 0.001), neurodevelopmental > healthy (*p* = 0.002), comorbidity > healthy (*p* < 0.001)]. In addition, analyses showed a stress-induced increase in subjective stress (*F*_(1, 190)_ = 13.71, *p* < 0.001) and heart rate (*F*_(1, 180)_ = 10.91, *p* = 0.001) for the combined subject group. While the subjective stress levels were higher in all three patient subgroups compared to the healthy subjects [stress-related > healthy (*p* < 0.001), neurodevelopmental > healthy (*p* = 0.002), comorbidity > healthy (*p* < 0.001)] , there were no group differences in heart rate related to stress induction (*F*_(3, 180)_ = 0.90, *p* = 0.441; Supplemental Table B).

### Functional MRI results

#### Networks of interest and results related to movement

The four networks of interest with the highest cross-correlation were selected (for the SN: *r* = 0.76, DMN: *r* = 0.54, left FPN: *r* = 0.60 and right FPN: *r* = 0.57). The selected networks included all areas that are typically considered as core regions in these networks. There were no significant differences between the subject subgroups related to the within network connectivity strength for our networks of interest, nor at baseline (SN: *F*_(3, 195)_ = 2.25, *p* = 0.084; DMN: *F*_(3, 195)_ = 1.76, *p* = 0.156; left FPN: *F*_(3, 195)_ = 1.31, *p* = 0.273; right FPN: *F*_(3, 195)_ = 1.82, *p* = 0.145) nor related to stress induced changes in connectivity strength (SN: *F*_(3, 195)_ = 0.82, *p* = 0.484; DMN: *F*_(3, 195)_ = 1.47, *p* = 0.223; left FPN: *F*_(3, 195)_ = 0.57, *p* = 0.636; right FPN: *F*_(3, 195)_ = 0.57, *p* = 0.636). There were no significant differences between the subject subgroups with respect to the mean relative framewise displacement in none of the three resting-state scans (resting-state scan 1: *F*_(3, 195)_ = 1.13, *p* = 0.338, resting-state scan 2: *F*_(3, 195)_ = 1.13, *p* = 0.339, resting-state scan 3: *F*_(3, 195)_ = 0.72, *p* = 0.541; Table 1).

#### Relationship between RNT and baseline connectivity

**Figure 3:**
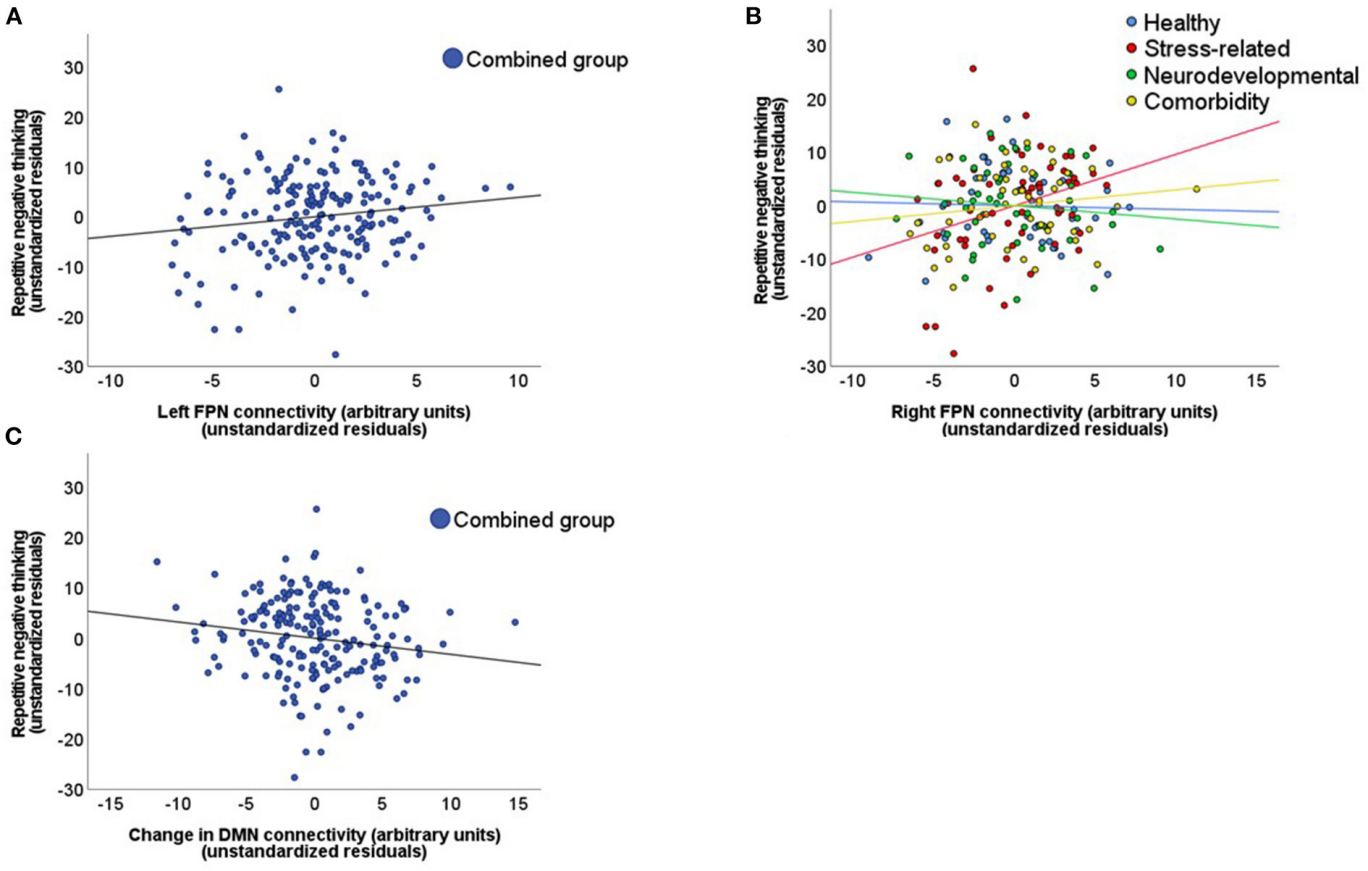
Relationship between repetitive negative thinking and network connectivity strength. These scatterplots display the relationship between repetitive negative thinking (RNT), measured with the PTQ sum score, and network connectivity strength. This relationship is displayed for: **(A)** The left frontoparietal network (FPN) in the combined subject group during the baseline resting-state scan. **(B)** The right FPN for the different subject subgroups during the baseline resting-state scan. **(C)** The stress induced changes in default mode network (DMN) connectivity in the combined subject group (stress minus neutral condition).

The hierarchical regression model in the combined subject group showed that the addition of the left FPN connectivity during resting-state scan 1 significantly improved the model fit (model 2 vs. model 1), and revealed a positive association between the left FPN connectivity strength and the PTQ sum score (β = 0.11, *p* = 0.042; Table 2, Figure 3A). This pattern did not differ between the different subject subgroups (*p* = 0.955) and *post-hoc* tests indicated that this effect was not driven by any specific PTQ subscale [“core characteristics” (β = 0.11, *p* = 0.050) “unproductiveness” (β = 0.11, *p* = 0.091), “capturing mental capacity” (β = 0.09, *p* = 0.120); Supplemental Table C]. RNT was not significantly associated with baseline connectivity of the SN (β = 0.00, *p* = 0.974) or DMN (β = −0.01, *p* = 0.913).

**Table 2:** Regression models: relationship between repetitive negative thinking (RNT) and baseline network connectivity strength.

	**Salience network**				**Default mode network**				**Left frontoparietal network**				**Right frontoparietal network**			
**Model summary**																
Model 1	*R*^2^ = 0.588, *F* change = 29.925, Sig. *F* change (*p* = ..) <0.001**				*R*^2^ = 0.588, *F* change = 29.925, Sig. *F* change (*p* = ..) <0.001**				*R*^2^ = 0.588, *F* change = 29.925, Sig. *F* change (*p* = ..) <0.001**				*R*^2^ = 0.588, *F* change = 29.925, Sig. *F* change (*p* = ..) <0.001**			
Model 2	*R*^2^ =0.588, *F* change = 0.001, Sig. *F* change (*p* = ..) = 0.974				*R*^2^ = 0.588, *F* change = 0.012, Sig. *F* change (*p* = ..) = 0.913				*R*^2^ = 0.597, *F* change = 4.195, Sig. *F* change (*p* = ..) = 0.042*				*R*^2^ = 0.592, *F* change = 1.941, Sig. *F* change (*p* = ..) = 0.165			
Model 3	*R*^2^ = 0.590, *F* change = 0.408, Sig. *F* change (*p* = ..) = 0.747				*R*^2^ = 0.596, *F* change = 1.204, Sig. *F* change (*p* = ..) = 0.310				*R*^2^ = 0.597, *F* change = 0.108, Sig. *F* change (*p* = ..) = 0.955				*R*^2^ = 0.609, *F* change = 2.761, Sig. *F* change (*p* = ..) = 0.043*			
**Full model results**	**Model 2**				**Model 2**				**Model 2**				**Model 3**			
	***B***	**SE *B***	***β***	***p*-value**	***B***	**SE *B***	***β***	***p*-value**	***B***	**SE *B***	***β***	***p*-value**	***B***	**SE *B***	***β***	***p*-value**
**Constant**	15.115	3.037		<0.001**	15.402	3.885		<0.001**	9.521	3.39		0.006**	12.533	3.949		0.002**
**Age (years)**	−0.015	0.048	−0.018	0.75	−0.017	0.049	−0.02	0.726	0.036	0.05	0.041	0.469	0.02	0.047	0.023	0.668
**Sex (male/female)**	−0.289	1.203	−0.012	0.81	−0.315	1.213	−0.013	0.795	−0.166	1.185	−0.007	0.889	−0.364	1.184	−0.015	0.759
**Level of education**															
No vs. middle	1.402	3.482	0.019	0.688	1.463	3.53	0.02	0.679	0.837	3.452	0.012	0.809	0.35	3.448	0.005	0.919
Low vs. middle	−0.715	2.047	−0.017	0.727	−0.701	2.049	−0.017	0.732	−0.78	2.024	−0.019	0.7	−0.713	2.048	−0.017	0.728
High vs. middle	0.413	1.328	0.016	0.756	0.421	1.327	0.017	0.752	0.385	1.308	0.015	0.769	0.246	1.316	0.01	0.852
**Subject group (dummy var)**															
SR	6.693	2.258	0.245	0.003**	6.663	2.27	0.244	0.004**	7.271	2.236	0.266	0.001**	1.444	4.127	0.053	0.727
ND	10.666	1.842	0.364	<0.001**	10.638	1.859	0.363	<0.001**	11.101	1.834	0.379	<0.001**	15.455	4.204	0.528	<0.001**
CM	11.337	2.153	0.398	<0.001**	11.314	2.156	0.397	<0.001**	11.871	2.13	0.417	<0.001**	9.715	4.164	0.341	0.021*
**IDS-SR**	0.417	0.053	0.546	<0.001**	0.417	0.053	0.546	<0.001**	0.409	0.052	0.536	<0.001**	0.43	0.052	0.563	<0.001**
**Within network connectivity**	−0.004	0.121	−0.002	0.974	−0.014	0.129	−0.006	0.913	0.39	0.191	0.112	0.042*	0.241	0.173	0.073	0.165
**Subject group x connectivity strength**																
SR vs. HC	N.S.				N.S.				N.S.				0.763	0.486	0.245	*p* = 0.120
ND vs. HC													−0.695	0.422	−0.278	*p* = 0.103
CM vs. HC													0.214	0.407	0.074	*p* = 0.601
SR vs. ND													1.429	0.503	0.594	*p* = 0.006**
CM vs. ND													0.831	0.418	0.423	*p* = 0.050
CM vs. SR													−0.477	0.463	−0.22	*p* = 0.305

*Dependent variable: PTQ sum score*.

*Model 1: predictors: constant, age, sex, level of education, subject group, IDS-SR*.

*Model 2: predictors from step 1 + baseline network connectivity strength*.

*Model 3: predictors from step 2 + interaction between subject group and baseline network connectivity strength*.

*CM: comorbidity group; HC: healthy controls; IDS-SR: Inventory of Depressive Symptomatology Self Report; ND: neurodevelopmental group; N.S.: no significant interaction; PTQ: Perseverative Thinking Questionnaire; Sig.: significant; SR: stress-related group; var: variables*.

*p < 0.05.

**p < 0.01.

Although the regression model that included the right FPN connectivity during resting-state scan 1 was not significantly improved by the addition of the right FPN connectivity strength (β = 0.07, *p* = 0.165; model 2 vs. model 1), the addition of the interaction terms did improve the model (*p* = 0.043; model 3 vs. model 2). This interaction was mainly driven by a difference in association with the PTQ sum score between the stress-related and neurodevelopmental group (β = 0.59, *p* = 0.006; Table 2, Figure 3B). The construction of the separate regression models for the four subgroups showed a positive relationship between the right FPN connectivity and the PTQ sum score for the stress-related group (β = 0.28, *p* = 0.042), which was absent in the neurodevelopmental group (β = −0.16, *p* = 0.182). *Post-hoc* tests revealed that the interaction effect between the stress-related and neurodevelopmental group was mainly driven by the PTQ subscales “unproductiveness” (β = 0.642, *p* = 0.005) and “capturing mental capacity” (β = 0.67, *p* = 0.003; Supplemental Table D).

#### Relationship between RNT and stress-induced connectivity changes

The hierarchical regression model in the combined subject group showed that the addition of stress-induced changes in DMN connectivity significantly improved the model fit (model 2 vs. model 1). The regression model revealed a negative association between the PTQ sum score and changes in DMN connectivity strength (β = −0.10, *p* = 0.029; Table 3, Figure 3C), indicating that a higher PTQ score was associated with a decrease in DMN connectivity after stress induction. This pattern did not differ between subject subgroups and *post-hoc* tests indicated that the main effect was strongest for the subscales “unproductiveness” (β = −0.11, *p* = 0.044) and “capturing mental capacity” (β = −0.11, *p* = 0.027; Supplemental Table E). There were no significant associations of RNT with stress-induced changes of the SN (β = 0.07, *p* = 0.162) or FPN (left FPN: β = −0.01, *p* = 0.916; right FPN: β = 0.08, *p* = 0.110).

**Table 3:** Regression models: relationship between repetitive negative thinking (RNT) and stress induced changes in network connectivity strength.

	**Salience network**				**Default mode network**				**Left frontoparietal network**				**Right frontoparietal network**			
**Model summary**																
**Model 1**	*R*^2^ = 0.588, *F* change = 29.925, Sig. *F* change (*p* = ..) <0.001**				*R*^2^ = 0.588, *F* change = 29.925, Sig. *F* change (*p* = ..) <0.001**				*R*^2^ = 0.588, *F* change = 29.925, Sig. *F* change (*p* = ..) <0.001**				*R*^2^ = 0.588, *F* change = 29.925, Sig. *F* change (*p* = ..) <0.001**			
**Model 2**	*R*^2^ = 0.592, *F* change = 1.969, Sig. *F* change (*p* = ..) = 0.162				*R*^2^ = 0.598, *F* change = 4.824, Sig. *F* change (*p* = ..) = 0.029*				*R*^2^ = 0.588, *F* change = 0.011, Sig. *F* change (*p* = ..) = 0.916				*R*^2^ = 0.593, *F* change = 2.583, Sig. *F* change (*p* = ..) = 0.110			
**Model 3**	*R*^2^ = 0.596, *F* change = 0.600, Sig. *F* change (*p* = ..) = 0.616				*R*^2^ = 0.609, *F* change = 1.684, Sig. *F* change (*p* = ..) = 0.172				*R*^2^ = 0.595, *F* change = 1.053, Sig. *F* change (*p* = ..) = 0.370				*R*^2^ = 0.597, *F* change = 0.620, Sig. *F* change (*p* = ..) = 0.603			
**Full model results**	**Model 2**				**Model 2**				**Model 2**				**Model 2**			
	***B***	**SE *B***	***β***	***p*-value**	***B***	**SE *B***	***β***	***p*-value**	***B***	**SE *B***	***β***	***p*-value**	**B**	**SE *B***	*β*	***p*-value**
**Constant**	15.115	3.037		<0.001**	15.402	3.885		<0.001**	9.521	3.39		0.006**	12.533	3.949		0.002**
**Age (years)**	−0.015	0.048	−0.018	0.75	−0.017	0.049	−0.02	0.726	0.036	0.05	0.041	0.469	0.02	0.047	0.023	0.668
**Sex (male/female)**	−0.289	1.203	−0.012	0.81	−0.315	1.213	−0.013	0.795	−0.166	1.185	−0.007	0.889	−0.364	1.184	−0.015	0.759
**Level of education**															
No vs. middle	1.402	3.482	0.019	0.688	1.463	3.53	0.02	0.679	0.837	3.452	0.012	0.809	0.35	3.448	0.005	0.919
Low vs. middle	−0.715	2.047	−0.017	0.727	−0.701	2.049	−0.017	0.732	−0.78	2.024	−0.019	0.7	−0.713	2.048	−0.017	0.728
High vs. middle	0.413	1.328	0.016	0.756	0.421	1.327	0.017	0.752	0.385	1.308	0.015	0.769	0.246	1.316	0.01	0.852
**Subject group (dummy var)**															
SR	6.693	2.258	0.245	0.003**	6.663	2.27	0.244	0.004**	7.271	2.236	0.266	0.001**	1.444	4.127	0.053	0.727
ND	10.666	1.842	0.364	<0.001**	10.638	1.859	0.363	<0.001**	11.101	1.834	0.379	<0.001**	15.455	4.204	0.528	<0.001**
CM	11.337	2.153	0.398	<0.001**	11.314	2.156	0.397	<0.001**	11.871	2.13	0.417	<0.001**	9.715	4.164	0.341	0.021*
**IDS–SR**	0.417	0.053	0.546	<0.001**	0.417	0.053	0.546	<0.001**	0.409	0.052	0.536	<0.001**	0.43	0.052	0.563	<0.001**
**Within network connectivity**	−0.004	0.121	−0.002	0.974	−0.014	0.129	−0.006	0.913	0.39	0.191	0.112	0.042*	0.241	0.173	0.073	0.165
**Subject group x connectivity strength**	N.S.				N.S.				N.S.				N.S.			

*Dependent variable: PTQ sum score*.

*Step 1: Predictors: constant, age, sex, level of education, subject group, IDS-SR*.

*Step 2: Predictors from step 1 + stress induced change in network connectivity strength*.

*Step 3: Predictors from step 2 + interaction between subject group and stress induced change in network connectivity strength*.

*CM: comorbidity group; IDS-SR: Inventory of Depressive Symptomatology Self Report; ND: neurodevelopmental group; N.S.: no significant interaction; PTQ: Perseverative Thinking Questionnaire; Sig.: significant; SR: stress-related group; var: variables*.

*p < 0.05.

**p < 0.01.

## Discussion

In this study, we investigated the relationship between RNT and network connectivity strength both at rest and in the aftermath of stress induction from a transdiagnostic perspective, by examining subjects across the psychopathological continuum from health to diverse non-psychotic psychiatric disorders. Our results showed a positive association between the level of RNT and FPN connectivity strength at rest. While this positive association was found across our whole sample for the left FPN, for the right FPN this relationship was only found in the stress-related disorders group. In addition, RNT was negatively associated with stress-induced changes in DMN connectivity. Below, we will discuss the significance of these findings. First, we will discuss the (transdiagnostic) associations at rest, before turning to the response to our experimentally well-controlled stressor.

Recently, Rosenbaum and colleagues (60) have shown that increased connectivity within the FPN at rest was related to high levels of rumination in healthy subjects. Our study extends these findings by showing transdiagnostic and disorder specific associations between RNT and the whole FPN. For the left FPN we found a positive association with RNT along the continuum from health to psychiatric disorders. For the right FPN this positive relationship was only present in the stress-related group and differed significantly from the neurodevelopmental group, which may relate to specific psychopathological patterns in the stress-related group. Notably, this pattern of the right FPN was driven by the “unproductiveness” and “capturing mental capacity” characteristics of RNT. Moreover, the analyses showed a higher unproductiveness-score on the RNT questionnaire for the stress-related group, than for the neurodevelopmental group. Interestingly, the unproductiveness of repetitive thinking is positively associated with the severity of psychopathology over and above the pure frequency of RNT (61).

There are several possible explanations for the positive association between the FPN connectivity strength and RNT. First, the FPN has been implicated in emotion regulation and top-down control (15). Thus, subjects who have a stronger (bottom-up) drive for negative thinking (higher trait RNT), may upregulate the FPN in an attempt to achieve top-down control (3, 8). Second, according to the impaired disengagement theory, subjects prone to RNT have impairments in exercising (top-down) attentional control to disengage from negative thoughts (8). Therefore, they may compensate for these impairments with stronger FPN connectivity. Third, the FPN may be involved more intrinsically in the higher-order cognitive aspects of RNT itself, e.g., in a (unproductive) problem-solving attempt (16, 29, 62). There may also be a lateralization effect, with a differential involvement of the left and right FPN, as is suggested by previous research (63).

Interestingly, neurostimulation studies provide evidence for a lateralization effect regarding the left and right FPN in RNT. In health, experimental stimulation of the left FPN with transcranial Direct Current Stimulation (tDCS) led to increased left prefrontal activity and a decrease in state RNT (64). Furthermore, the clinical non-invasive neurostimulation literature has mainly studied worry in generalized anxiety disorder (GAD) (65) and rumination in depression (6). Treatment studies in depression show that depression and rumination can be relieved by both low-frequency repetitive Transcranial Magnetic Stimulation (rTMS) applied over the right dorsolateral PFC (FPN), which is known to inhibit this area, and high frequency rTMS applied over the left dorsolateral PFC, that activates this region (66). Interestingly, also in GAD the inhibition of core regions of the right FPN (65, 67), with low-frequency rTMS, led to a decrease in worry severity and increased remission rates. Together with our results, these findings may support a lateralization effect with adaptive and maladaptive involvement of the left and right FPN in RNT respectively.

Although earlier studies found a negative association between RNT and connectivity within the DMN at rest (29, 30), we did not find this relationship. This difference in findings, may be related to differences in analysis methods, since these earlier studies used seed-based methods (29, 30), while we investigated the connectivity strength within the DMN as a whole. Another important difference is that we studied content-independent RNT in a transdiagnostic sample, while these earlier studies investigated worry in GAD (30) and rumination in depression and health (29). Interestingly, while we did not find significant results for the DMN at rest, our experimental stressor did reveal a negative relationship between the DMN and RNT.

Our DMN result emphasizes that stress induction may unmask specific vulnerabilities to stress. The negative relationship between RNT and stress-induced changes in DMN connectivity is in line with a recent study (68), showing a negative association between RNT and within DMN connectivity in response to sad-mood induction in health and remitted depression. These results may be related to the fact that stronger DMN connectivity is associated with a more flexible pattern of mind wandering, while RNT is characterized by an inflexible and repetitive thinking style (29, 69).

Although previous studies have shown an important role for the SN in RNT (2, 25), we did not find any significant results for this network. The absence of significant results for the SN possibly results from the static measure of mean within network connectivity that we investigate. The role of the SN in RNT can possibly be better studied by investigating the between network connectivity with other networks (2) or the more dynamic and flexible nature in which it facilitates switching between the DMN and FPN (14, 25).

While in clinical practice patients are generally classified, using heterogeneous psychiatric classifications (70), here we want to illustrate how our understanding of psychopathology may be improved by integrating a dimensional approach to RNT, with the impaired disengagement theory and the stress-vulnerability model. From a dimensional perspective, common traits of neurodevelopmental disorders can be seen as a vulnerability factor for developing RNT under stress. For example, the degree of cognitive inhibition of a person can be seen as a continuous trait, with impaired cognitive inhibition (common in ADHD) at the extreme of this spectrum (11). When confronted with a stressor, impaired cognitive inhibition serves as a vulnerability factor for developing RNT, due to impaired disengagement from negative thoughts (8), which in turn may lead to more stress-related symptoms (5). Our understanding of psychopathology could be improved by future studies applying such a dimensional, transdiagnostic conceptualization of how symptoms result from the interaction between stressors and specific traits.

The main strength of our study is that this is the first study investigating the neurobiological mechanisms of RNT transdiagnostically both at rest and related to an experimentally well-controlled stress induction procedure. However, our study has to be interpreted in light of some limitations. First, although we hypothesize that there may be a lateralization effect regarding the adaptive and maladaptive roles of the FPNs in RNT, the cross-sectional nature of our study does not allow us to make causal inferences about this with certainty. Future prospective and MRI neurostimulation studies should further investigate this hypothesis. Second, the PTQ-score measures trait RNT and we did not measure state RNT during the MRI scan itself. However, a recent study found that state and trait rumination were only moderately correlated (29). As trait RNT has been identified as an important risk factor for various psychiatric disorders, we think it is important to study this construct in relation to the resting-state and stress.

Taken together, our results provide initial insight in the neural mechanisms of RNT across the psychopathological continuum. The positive association between left FPN connectivity and RNT at rest, across the entire sample, may reflect the need for upregulation of the FPN for top-down control of negative thoughts. The negative association between RNT and changes in DMN connectivity, which were revealed by stress, may reflect a less flexible and more repetitive thinking style under stress. Importantly, RNT is a promising treatment target (71), with non-invasive neurostimulation treatments directed at the FPN showing positive effects in psychiatric disorders in which RNT is a core feature (65, 72). A better understanding of the neural mechanisms of RNT may improve future treatments, by helping to develop neural circuit-guided personalized treatments (73).

## Data availability statement

The preprocessed MRI data is available on request in line with the institutional ethics guidelines. Requests to access the datasets should be directed to Jasper van Oort, jasper.vanoort@radboudumc.nl.

## Ethics statement

The MIND-Set study has been approved by the Ethical Review Board of the Radboudumc (Nijmegen, the Netherlands) and all participants signed informed consent forms before participation.

## Author contributions

All authors listed have made a substantial, direct, and intellectual contribution to the work and approved it for publication.

## Conflict of interest

The authors declare that the research was conducted in the absence of any commercial or financial relationships that could be construed as a potential conflict of interest.

## Publisher's note

All claims expressed in this article are solely those of the authors and do not necessarily represent those of their affiliated organizations, or those of the publisher, the editors and the reviewers. Any product that may be evaluated in this article, or claim that may be made by its manufacturer, is not guaranteed or endorsed by the publisher.
