# Supplementary material for: Neural correlates of repetitive negative thinking: Dimensional evidence across the psychopathological continuum
Source: Front Psychiatry. 2022 Jul 22;13:915316. doi: 10.3389/fpsyt.2022.915316 (PMC9356323; doi:10.3389/fpsyt.2022.915316)
Supplement: Supplementary file 1 [file Data_Sheet_1.pdf]

Supplemental Table A. Correlations between the IDS and PTQ in the combined subject group

	Combined subject group (n = 199)	Healthy controls (n = 46)	Stress-related group (n = 57)	Neurodevelopmental group (n = 46)	Comorbidity group (n = 50)
**IDS-SR**					
**PTQ sum score**	r = 0.696, p < 0.001**	r = 0.478, p = 0.001**	r = 0.494, p < 0.001**	r = 0.661, p < 0.001**	r = 0.469, p = 0.001**
**PTQ subscale core characteristics**	r = 0.682, p < 0.001**	r = 0.479, p = 0.001**	r = 0.485, p < 0.001**	r = 0.698, p < 0.001**	r = 0.455, p = 0.001**
**PTQ subscale unproductiveness**	r = 0.594, p < 0.001**	r = 0.431, p = 0.003**	r = 0.378, p = 0.004**	r = 0.364, p = 0.013*	r = 0.232, p = 0.104
**PTQ subscale capturing mental capacity**	r = 0.654, p < 0.001**	r = 0.321, p = 0.030*	r = 0.508, p < 0.001**	r = 0.446, p = 0.002**	r = 0.506, p < 0.001**

Abbreviations: IDS-SR: Inventory of Depressive Symptomatology Self Report, PTQ: Perseverative Thinking Questionnaire.
* p < 0.05, ** p < 0.01

Supplemental Table B. Behavioral and physiological measures during the experiment.

	Combined subject group (n = 199)	Healthy controls (n = 46)	Stress-related group (n = 57)	Neurodevelopmental group (n = 46)	Comorbidity group (n = 50)	(repeated measures) AN(C)OVA to test for differences for the four subgroups in the combined subject group (F, p-value)	Post-hoc comparisons between the four subgroups, if AN(C)OVA is significant (statistics shown if p < 0.05) (p-value)
**Stress levels**							
**Baseline measures (mean, SD)**							
Heart rate (BPM) †	66.08 (± 10.55)	63.38 (± 8.85)	66.99 (± 11.39)	66.24 (± 10.97)	67.38 (± 10.51)	F(3,183) = 1.16, p = 0.327	
Subjective stress	3.24 (± 2.26)	1.89 (± 1.74)	3.81 (± 2.15)	3.09 (± 2.14)	3.96 (± 2.41)	F(3,190) = 10.42, p < 0.001**	SR > HC (p < 0.001**), ND > HC (p = 0.002**), CM > HC (p < 0.001**), CM > ND (p = 0.046*)
**Stress induced changes in stress level (mean, SD)**							
Heart rate (BPM) †	NeuM: 65.82 (± 10.76) AvM: 67.80 (11.25)	NeuM: 63.10 (± 9.14) AvM: 65.48 (± 10.08)	NeuM: 66.14 (± 11.12) AvM: 67.74 (± 11.69)	NeuM: 66.13 (± 11.99) AvM: 68.44 (± 11.60)	NeuM: 67.60 (± 10.44) AvM: 69.37 (± 11.40)	- Time effect: F(1,180) = 10.91, p = 0.001**	AvM > NeuM
						- Group effect: F(3,180) = 0.90, p = 0.441	
						- Time by group interaction: F(3,180) = 0.62, p = 0.602	
Subjective stress ‡	neutral: 2.91 (± 2.15) stress: 4.80 (± 2.66)	neutral 1.57 (± 1.57) stress: 3.59 (± 2.71)	neutral : 3.49 (± 2.11) stress: 5.32 (± 2.53)	neutral : 2.78 (± 2.11) stress: 4.65 (± 2.65)	neutral : 3.62 (± 2.17) stress: 5.46 (± 2.43)	- Time effect: F(1,190) = 13.71, p < 0.001**	Stress > neutral
						- Group effect: F(3,190) = 9.81, p < 0.001**	SR > HC (p < 0.001**), ND > HC (p = 0.002**), CM > HC (p < 0.001**)
						- Time by group interaction: F(3,190) = 0.16, p = 0.921	

Abbreviations: AvM: during aversive movie, BPM: beats per minute, CM: comorbidity group, HC: healthy controls, ND: neurodevelopmental group, NeuM: during neutral movie, PTQ: Perseverative Thinking Questionnaire, SR: stress-related group.

† Number of subjects is lower than the total subject group, due to missing data. See van Oort et al. 2020 for information with respect to missing data.

‡ neutral: is subjective stress score after the neutral movie, stress: subjective stress score after the aversive movie

**p* < 0.05, ** p < 0.01

Supplemental Table C. Post-hoc regression models for the relationship between Perseverative Thinking Questionnaire (PTQ) subscales and baseline left frontoparietal network (FPN) connectivity strength in the combined subject group.

	Core characteristics				Unproductiveness				Capturing mental capacity			
**Model summary**												
**Model 1**	R^2 = 0.562, F change = 26.905, Sig. F change (p = ..) < 0.001**				R^2 = 0.430, F change = 15.843, Sig. F change (p = ..) < 0.001**				R^2 = 0.568, F change = 27.667, Sig. F change (p = ..) < 0.001**			
**Model 2**	R^2 = 0.571, F change = 3.907, Sig. F change (p = ..) = 0.050				R^2 = 0.439, F change = 2.884, Sig. F change (p = ..) = 0.091				R^2 = 0.574, F change = 2.435, Sig. F change (p = ..) = 0.120			
**Model 3**	R^2 = 0.572, F change = 0.160, Sig. F change (p = ..) = 0.923				R^2 = 0.442, F change = 0.407, Sig. F change (p = ..) = 0.748				R^2 = 0.579, F change = 0.702, Sig. F change (p = ..) = 0.552			
**Full model results**	**Model 2**				**Model 2**				**Model 2**			
	**B**	**SE B**	**β**	**p**	**B**	**SE B**	**β**	**p**	**B**	**SE B**	**β**	**p**
**Constant**	7.042	2.158		0.001**	1.727	0.851		0.044*	0.752	0.791		0.342
**Age (years)**	0.013	0.032	0.024	0.680	0.008	0.012	0.041	0.542	0.015	0.012	0.077	0.186
**Sex (male/female)**	-0.396	0.754	-0.026	0.600	0.079	0.297	0.015	0.791	0.151	0.276	0.027	0.585
**Level of education**												
**No vs middle**	1.250	2.197	0.028	0.570	-0.937	0.867	-0.061	0.281	0.524	0.805	0.032	0.516
**Low vs middle**	-0.692	1.288	-0.027	0.592	-0.021	0.508	-0.002	0.967	-0.067	0.472	-0.007	0.888
**High vs middle**	0.490	0.832	0.032	0.557	0.168	0.328	0.031	0.610	-0.273	0.305	-0.048	0.373
**Subject group (dummy var)**												
**SR**	3.309	1.423	0.196	0.021*	2.233	0.561	0.384	< 0.001**	1.729	0.521	0.279	0.001**
**ND**	6.241	1.167	0.346	< 0.001**	2.047	0.460	0.328	< 0.001**	2.812	0.428	0.423	< 0.001**
**CM**	6.484	1.355	0.369	< 0.001**	2.440	0.535	0.403	< 0.001**	2.947	0.497	0.456	< 0.001**
**IDS-SR**	0.266	0.033	0.565	< 0.001**	0.061	0.013	0.378	< 0.001**	0.082	0.012	0.471	< 0.001**
**Left FPN connectivity strength**	0.240	0.121	0.111	0.050	0.081	0.048	0.109	0.091	0.069	0.044	0.088	0.120
**Subject group x connectivity strength**	**N.S.**				**N.S.**				**N.S.**			

Abbreviations: CM: comorbidity group, FPN: frontoparietal network, ND: neurodevelopmental group, N.S.: no significant interaction, SR: stress-related group, var: variables.
*p < 0.05, ** p < 0.01

Supplemental Table D. Post-hoc regression models for the relationship between Perseverative Thinking Questionnaire (PTQ) subscales and baseline right frontoparietal network (FPN) connectivity strength in the combined subject group.

	Core characteristics				Unproductiveness				Capturing mental capacity			
**Model summary**												
**Model 1**	R^2 = 0.562, F change = 26.905, Sig. F change (p = ..) < 0.001**				R^2 = 0.430, F change = 15.843, Sig. F change (p = ..) < 0.001**				R^2 = 0.568, F change = 27.667, Sig. F change (p = ..) < 0.001**			
**Model 2**	R^2 = 0.567, F change = 2.224, Sig. F change (p = ..) = 0.138				R^2 = 0.432, F change = 0.600, Sig. F change (p = ..) = 0.440				R^2 = 0.571, F change = 1.166, Sig. F change (p = ..) = 0.282			
**Model 3**	R^2 = 0.578, F change = 1.658, Sig. F change (p = ..) = 0.178				R^2 = 0.455, F change = 2.612, Sig. F change (p = ..) = 0.053				R^2 = 0.595, F change = 3.649, Sig. F change (p = ..) = 0.014*			
**Full model results**	Model 2				Model 3				Model 3			
	B	SE B	β	p	B	SE B	β	p	B	SE B	β	p
**Constant**	8.323	1.931		< 0.001**	2.468	0.993		0.014*	1.485	0.912		0.105
**Age (years)**	0.001	0.030	0.001	.981	0.003	0.012	0.015	0.808	0.013	0.011	0.065	0.240
**Sex (male/female)**	-0.452	0.756	-0.029	.551	0.033	0.298	0.006	0.912	0.111	0.273	0.020	0.685
**Level of education**												
**No vs middle**	1.463	2.201	0.033	.507	-1.033	0.867	-0.067	0.235	0.315	0.796	0.019	0.692
**Low vs middle**	-0.706	1.294	-0.028	.586	0.027	0.515	0.003	0.958	-0.094	0.473	-0.010	0.843
**High vs middle**	0.431	0.838	0.028	.607	0.155	0.331	0.029	0.640	-0.317	0.304	-0.055	0.298
**Subject group (dummy var)**												
**SR**	3.168	1.425	0.188	.027*	0.869	1.037	0.149	0.403	0.217	0.953	0.035	0.821
**ND**	6.149	1.170	0.340	< 0.001**	3.197	1.057	0.513	0.003**	3.892	0.971	0.586	< 0.001**
**CM**	6.416	1.362	0.365	< 0.001**	2.125	1.047	0.351	0.044*	1.989	0.962	0.308	0.040*
**IDS-SR**	0.271	0.033	0.575	< 0.001**	0.066	0.013	0.406	< 0.001**	0.087	0.012	0.501	< 0.001**
**Right FPN connectivity strength**	0.164	0.110	0.080	0.138	0.034	0.043	0.048	0.440	0.043	0.040	0.058	0.282
**Subject group x connectivity strength**												
**SR vs HC**	**N.S.**				0.181	0.121	0.261	0.139	0.197	0.104	0.295	0.062
**ND vs HC**					-0.184	0.109	-0.359	0.096	-0.171	0.109	-0.288	0.122
**CM vs HC**					0.024	0.106	0.041	0.822	0.106	0.092	0.159	0.256
**SR vs ND**					0.354	0.124	0.642	0.005**	0.359	0.116	0.670	0.003**
**CM vs ND**					0.178	0.106	0.416	0.096	0.254	0.103	0.538	0.016*
**CM vs SR**					-0.134	0.116	-0.273	0.250	-0.077	0.101	-0.158	0.446

Abbreviations: CM: comorbidity group, FPN: frontoparietal network, HC: healthy controls, ND: neurodevelopmental group, N.S.: no significant interaction, SR: stress-related group, var: variables.
*p < 0.05, ** p < 0.01

Supplemental Table E. Post-hoc regression models for the relationship between the Perseverative Thinking Questionnaire (PTQ) subscales and stress induced changes in default mode network (DMN) connectivity in the combined subject group.

	Core characteristics				Unproductiveness				Capturing mental capacity			
**Model summary**												
**Model 1**	R^2 = 0.562, F change = 26.905, Sig. F change (p = ..) < 0.001**				R^2 = 0.430, F change = 15.843, Sig. F change (p = ..) < 0.001**				R^2 = 0.568, F change = 27.667, Sig. F change (p = ..) < 0.001**			
**Model 2**	R^2 = 0.569, F change = 3.354, Sig. F change (p = ..) = 0.069				R^2 = 0.442, F change = 4.119, Sig. F change (p = ..) = 0.044*				R^2 = 0.580, F change = 4.997, Sig. F change (p = ..) = 0.027*			
**Model 3**	R^2 = 0.581, F change = 1.647, Sig. F change (p = ..) = 0.180				R^2 = 0.461, F change = 2.120, Sig. F change (p = ..) = 0.099				R^2 = 0.584, F change = 0.663, Sig. F change (p = ..) = 0.576			
**Full model results**	**Model 2**				**Model 3**				**Model 2**			
	**B**	**SE B**	**B**	**p**	**B**	**SE B**	**β**	**p**	**B**	**SE B**	**β**	**p**
**Constant**	10.228	1.314		< 0.001**	2.796	0.520		< 0.001**	1.642	0.478		0.001**
**Age (years)**	-0.017	0.027	-0.032	0.535	-0.003	0.011	-0.014	0.810	0.007	0.010	0.034	0.495
**Sex (male/female)**	-0.417	0.755	-0.027	0.581	0.061	0.294	0.012	0.835	0.154	0.274	0.027	0.575
**Level of education**												
**No vs middle**	1.135	2.207	0.025	0.608	-1.182	0.871	-0.077	0.177	0.420	0.802	0.026	0.602
**Low vs middle**	-0.612	1.290	-0.024	0.636	0.204	0.512	0.023	0.691	-0.038	0.469	-0.004	0.936
**High vs middle**	0.345	0.838	0.022	0.681	0.072	0.328	0.013	0.827	-0.339	0.305	-0.059	0.267
**Subject group (dummy var)**												
**SR**	3.240	1.422	0.192	0.024*	2.297	0.564	0.395	< 0.001**	1.752	0.517	0.283	0.001**
**ND**	6.196	1.167	0.343	< 0.001**	2.085	0.464	0.335	< 0.001**	2.834	0.424	0.426	< 0.001**
**CM**	6.403	1.354	0.365	< 0.001**	2.527	0.536	0.417	< 0.001**	2.962	0.492	0.458	< 0.001**
**IDS-SR**	0.270	0.033	0.574	< 0.001**	0.061	0.013	0.374	< 0.001**	0.083	0.012	0.478	< 0.001**
**Stress induced change in DMN connectivity strength**	-0.170	0.093	-0.090	0.069	-0.074	0.037	-0.113	0.044*	-0.076	0.034	-0.108	0.027*
**Subject group x connectivity strength**												
**SR vs HC**	**N.S.**				0.052	0.120	0.051	0.663	**N.S.**			
**ND vs HC**					0.107	0.106	0.125	0.313				
**CM vs HC**					-0.123	0.098	-0.142	0.216				
**SR vs ND**					-0.060	0.115	-0.074	0.603				
**CM vs ND**					-0.269	0.091	-0.424	0.004**				
**CM vs SR**					-0.165	0.101	-0.218	0.107				

Abbreviations: CM: comorbidity group, DMN: default mode network, ND: neurodevelopmental group, N.S.: no significant interaction, SR: stress-related group, var: variables. *p < 0.05, ** p < 0.01
